# Setting the Balance between the Lexical and Sublexical Pathways of Dual-Route Models of Reading: Insight from Atypical Dyslexia in Surgical Glioma Patients

**DOI:** 10.3389/fpsyg.2016.01730

**Published:** 2016-11-08

**Authors:** Emmanuel Mandonnet, Hugues Duffau

**Affiliations:** ^1^Neurosurgery, Lariboisière HospitalParis, France; ^2^Université Paris 7 DiderotParis, France; ^3^IMNC UMR 8165Orsay, France; ^4^Neurosurgery, Hôpital Gui de Chauliac, CHU MontpellierMontpellier, France; ^5^Institut National de la Santé et de la Recherche Médicale U1051Montpellier, France; ^6^Université Montpellier 2Montpellier, France

**Keywords:** computational models, reading, dual-route cascade, connectionist dual-process, glioma, surgery, fMRI

Reading is a complex skill that has been acquired only recently in the evolution of humans. This fascinating ability has motivated many neuropsychological models. In particular, it has been proposed that reading is sustained by two parallel and complementary systems (see for example Epelbaum et al., 2008 for a plausible anatomical implementation of this model): a lexico-semantic one and a phonological one (that further requires attentional resources for processing serially the different syllables of a given word). In parallel to advances in neuropsychological models of reading, computational models have been intensively studied. Such models attempt to go further than the usual “boxology”, by providing a simulation on a computer of the different cognitive processes that are involved in reading. One of the very first version in 1989 (Seidenberg and McClelland, 1989) was a connectionist approach, known as the “triangle model”, that already implemented the two routes (a semantic one and a phonological one). Since then, two categories have been competing in order to achieve the best fit between simulations and human datas in healthy and brain-damaged individuals: the dual-route cascade (DRC) (Coltheart et al., 2001) and the connectionist dual-process (CDP) (Zorzi et al., 1998) and its updated versions CDP+ (Perry et al., 2007), CDP++ (Perry et al., 2010), and CDP++-parser (Perry et al., 2013). Both kind of models share the same architecture regarding the lexical route, while differing in the sublexical route. In both cases, the balance between the lexical and sub-lexical route is a critical issue: whenever the lexical route is too strong, pseudowords will be read as a lexical neighbor, and whenever the sub-lexical route is too strong, error rates rise for irregular words (which are regularized by the sublexical route). As clearly explained in the paragraph “Searching parameter space” of Coltheart et al. (2001), the optimal balance is found in the DRC model by trial and error, testing the ability of the system to read a specific pair of irregular word and pseudoword (respectively “chef” and “starn”). Similarly, it is stated in Perry et al. (2007): “The first step was to determine the appropriate balance between lexical and sublexical phonology, which in turn largely depends on the speed at which the serial process of grapheme parsing occurs. These parameters need to be chosen together, because slower grapheme parsing speeds reduce the amount of sublexical phonology in the model, and faster speeds increase it. Performance on irregular words provides a particularly important benchmark for parameter setting.” Importantly, in both models, there is no explicit computational system that resets the balance between the two routes depending on the characteristics of each trial (regular, irregular, and pseudowords).

In a recent paper, Zemmoura et al. focused on reading abilities in a small series of patients operated on in awake conditions for a diffuse low-grade glioma of the posterior temporo-basal region (Zemmoura et al., 2015). Most importantly, it was reported that some patients (# 3, 4, & 5) had a long term impairment for reading irregular words and pseudo-words, while reading regular words was spared. These three patients had a cavity extending in the short vertical indirect portion of the arcuate fasciculus (see Catani et al., 2005 for a description of this tract).

This new kind of dissociation differs from the commonly reported surface dyslexia (specific deficit in irregular words reading) and phonological dyslexia (selective difficulty for reading pseudo-words). Those two acquired dyslexia are easily interpreted in the dual-route models: the lexical pathway is damaged in the surface dyslexia, while the sublexical pathway is injured in the phonological dyslexia. Our atypical dyslexia is more difficult to understand within the framework of dual-route processes. Zemmoura et al. (2015) thus interpreted these datas by assigning a feedback role to this short vertical indirect portion of the arcuate fasciculus: “Thus, to explain that reading out loud irregular and pseudowords can be impaired by an unique lesion, we propose that reading these words do not rely on a simple serial, feed-forward neural system, but rather on feedback connections linking visual to nonvisual information to create an interactive system for visual words recognition (Twomey et al., 2011). According to our observations, these feedback connections might therefore be involved in both the semantic and the phonological pathways. More particularly, this interactive system should be mostly recruited when spelling-sound incoherence (the case for irregular words) or when absence of meaning (the case of pseudowords) is detected;…”.

The simplest way to implement such feedback is the following (see Figure 1): let us assume that the two routes operate independently (at a subconscious level), each leading to a preliminar response in a (subliminal) phonological buffer. Then, some part of the brain analyzes the difference between the phonological responses generated by the lexical route and the sublexical route, and this signal is used as a feedback to re-balance the contribution of each route. In case of regular words, this difference is null, and no feedback is needed that would favor either routes. In case of pseudo-words, the difference is said negative, as there is no response from the lexical pathway. The feedback reinforces the sublexical pathway and inhibits the re-entry in the lexical pathway. In case of irregular words, the difference is said positive, as there is a difference and the phonological output coming from the lexical pathway is not zero. This “positive signal” will feedback in order to inhibit the sublexical route and amplify the lexical one. Within this “boxology” model, one can easily understand that an absence of this feeback signal will impact irregular and pseudo-words reading, while leaving unaffected regular words reading.

**Figure 1:**
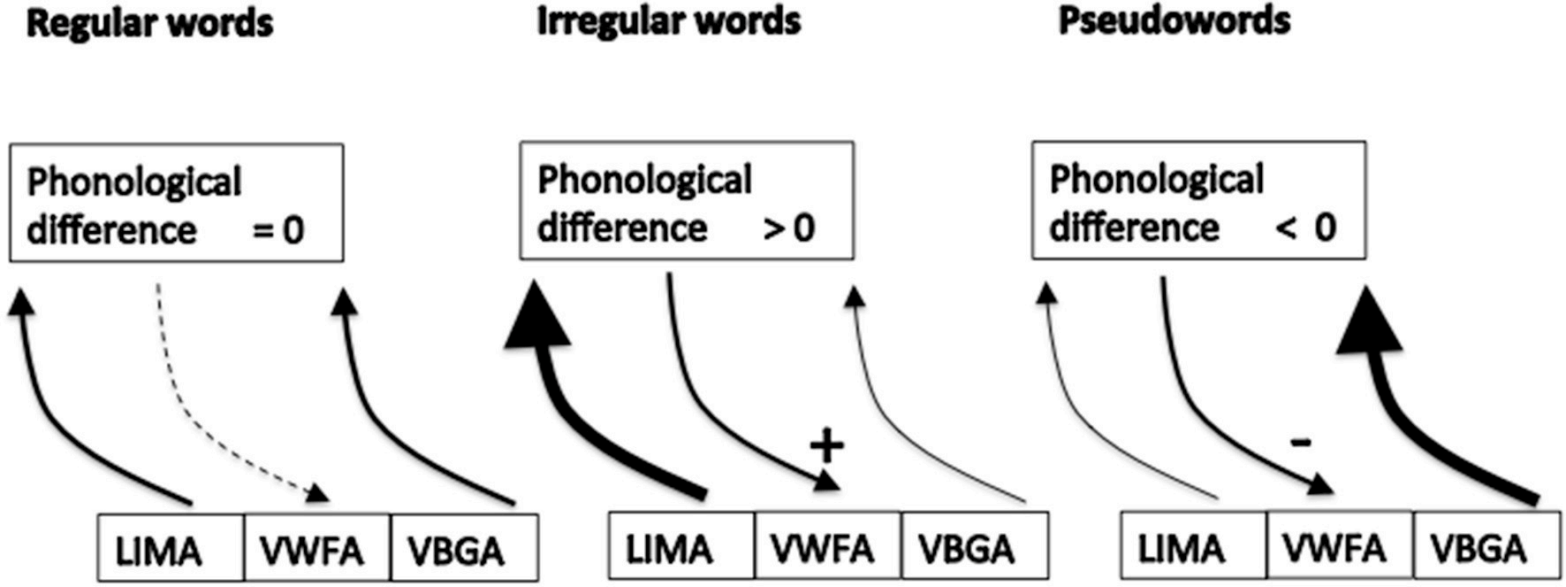
**“Boxology” model of a feedback allowing to tune the balance between the lexical and sub-lexical routes**. LIMA, lateral inferotemporal multimodal area; VWFA, visual word form area; VBGA, visual bigram area. For regular words, the preliminar phonological outputs from the lexical and sublexical routes are the same, feedback signal is unnecessary. For irregular words, the difference between the two routes is said positive, generating a feedback signal boosting the lexical route while stopping the sublexical one. For pseudowords, the difference is said negative (as there is no entry from the lexical route), resulting in a feedback signal inhibiting any re-entry in the lexical route.

From previous neurospychological models and Zemmoura's work, one can propose the following anatomical substrates for this model: the visual bigram area (VBGA) (Dehaene et al., 2005) directly feeds the sublexical route, allowing the serial process of phonological reading, thanks to the participation of parietal areas sustaining the required attentional resources. Through connections corresponding anatomically to the U-fibers of the occipito-temporal projection system (Catani et al., 2003), this VBGA also feeds the visual word form area (VWFA) (Dehaene et al., 2005), which is itself linked to the lateral inferotemporal multimodal area (LIMA) (Cohen et al., 2004). This latter LIMA can be considered as the system making the association between the orthographic and phonological lexicons within the lexical route. The phonological buffer that performs the analysis of the difference between the outputs of the two routes is not well localized anatomically, but likely involves the superior temporal gyrus, the supramarginal gyrus, and the posterior part of inferior frontal gyrus (Epelbaum et al., 2008), all areas known to be involved in phonological processing (Vigneau et al., 2006). Finally, the feedback signal is sent through the short vertical portion of the arcuate fasciculus, amplifying VWFA, and its input to the LIMA [i.e., the interactive activation lexical network of Perry et al. (2007)] for irregular words, and inhibiting this same network in case of pseudowords reading (hence favoring the sublexical route). Finally, the lexical route is also probably in close relationship with the semantic system (as in the triangle model), but this interaction is beyond the scope of this paper.

Furthermore, this model could provide a new basis for analyzing functional MRI studies of reading. We would expect that areas computing the phonological difference and sending the feedback signal would show up in the contrast condition of irregular words or pseudowords versus regular words, and wash out in the contrast condition of irregular words versus pseudowords. Reinterpreting in this way data from a previous study (Binder et al., 2005), one can identify those areas as a network comprising Broca's area on both sides, pre-SMA on both sides, and part of the left supramarginal and angular gyri (see Figures 4B,C in the aforementioned paper). Otherwise, the LIMA should show an activity increasing in the order pseudowords < regular words < irregular words, an hypothesis that could be tested in future studies.

Although we found this “boxology” model of feedback very convenient to explain the atypical pattern of selective dyslexia for irregular and pseudo-words as reported by Zemmoura et al. (2015), it remains to be demonstrated whether these results could play any role in refining computational models of reading. This leads us to ask to the community of computational modelers the following questions:

- is it possible to simulate the atypical dyslexia within the current framework of DRC and CDP++-parser?
- if yes, which parameters should be changed and how?
- if not, does it mean that an explicit feedback (as the one we qualitatively described) should be incorporated in the models?

These questions should motivate further work in the exciting field of computational models of reading.

## Author contributions

All authors listed, have made substantial, direct and intellectual contribution to the work, and approved it for publication.

### Conflict of interest statement

The authors declare that the research was conducted in the absence of any commercial or financial relationships that could be construed as a potential conflict of interest.
